# Supplementary material for: Honokiol and Alpha-Mangostin Inhibit Mayaro Virus Replication through Different Mechanisms
Source: Molecules. 2022 Oct 29;27(21):7362. doi: 10.3390/molecules27217362 (PMC9659048; doi:10.3390/molecules27217362)
Supplement: Supplementary file 1 [file molecules-27-07362-s001.zip › molecules-1899674-supplementary.pdf]

molecules

MDPI

Supplementary material

Honokiol and Alpha-Mangostin Inhibit Mayaro Virus Replication through Different Mechanisms

Patricia Valdés-Torres [1,2†], Dalkiria Campos [1†], Madhvi Bhakta [1], Paola Elaine Galán-Jurado [1], Armando A. Durant-Archibold [3], and JoséGonzález-Santamaría [1,*]

[1] Grupo de Biología Celular y Molecular de Arbovirus, Instituto Conmemorativo Gorgas de Estudios de la Salud, Panama 0816-02593, Panama

[2] Programa de Maestría en Microbiología Ambiental, Universidad de Panamá, Panama 0824, Panama

[3] Departamento de Bioquímica, Facultad de Ciencias Naturales, Exactas y Tecnología, Universidad de Panamá, Panama 0824-03366, Panama

* Correspondence: jgonzalezsantamaria@gorgas.gob.pa; Tel.: +507 527-4814

† These authors contributed equally to this work.

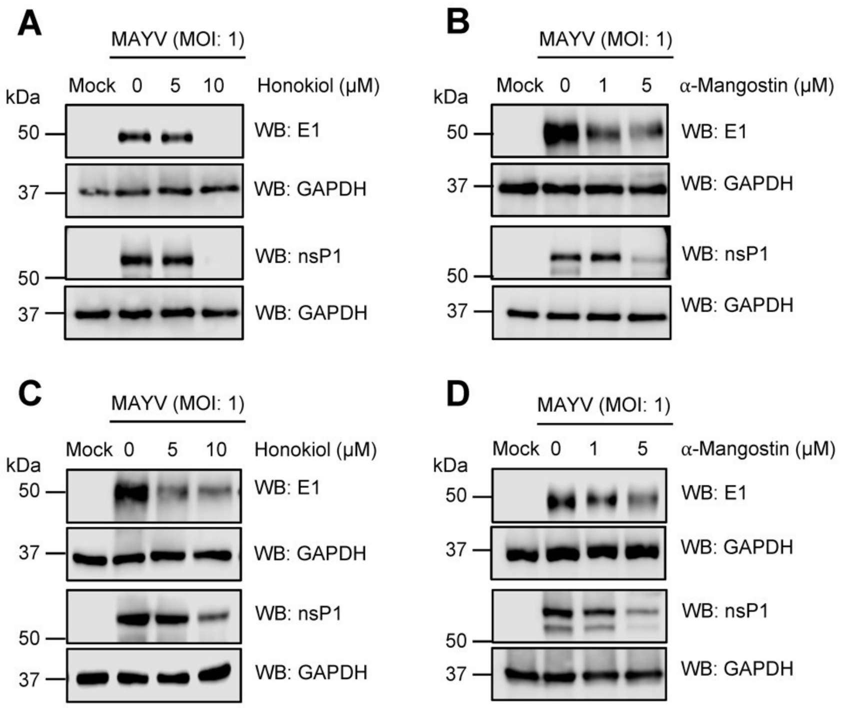

Figure S1. Honokiol and α-Mangostin promote a reduction in the expression of MAYV E1 and nsP1 proteins in Vero-E6 cells and HDFs. Vero-E6 cells (**A, B**) and HDFs (**C, D**) were infected with MAYV AVR0565 strain at an MOI of 1 and then treated with Honokiol (**A**) or α-Mangostin (**B**) at the indicated doses. After 24h of incubation, protein extracts were obtained and E1 and nsP1 viral protein levels were analyzed using Western blot. GAPDH protein was used as a loading control. kDa: kilodaltons; WB: Western blot.
